# Silicon Enhances Biomass and Grain Yield in an Ancient Crop Tef [*Eragrostis tef (Zucc.) Trotter*]

**DOI:** 10.3389/fpls.2020.608503

**Published:** 2020-11-26

**Authors:** Ayalew Ligaba-Osena, Wanli Guo, Sang Chul Choi, Matthew Alan Limmer, Angelia L. Seyfferth, Bertrand B. Hankoua

**Affiliations:** ^1^Laboratory of Molecular Biology and Biotechnology, Department of Biology, The University of North Carolina at Greensboro, Greensboro, NC, United States; ^2^Department of Biotechnology, College of Life Sciences and Medicine, Zhejiang Sci-Tech University, Hangzhou, China; ^3^Department of Plant and Soil Sciences, The University of Delaware, Newark, DE, United States; ^4^Plant Biotechnology Lab, Department of Agriculture and Natural Resources, College of Agriculture, Sciences and Technology, Delaware State University, Dover, DE, United States

**Keywords:** *Eragrostis tef*, silicon, biomass, grain yield, silicon transporter, mineral content

## Abstract

Silicon (Si) is one of the beneficial plant mineral nutrients which is known to improve biotic and abiotic stress resilience and productivity in several crops. However, its beneficial role in underutilized or “orphan” crop such as tef [*Eragrostis tef (Zucc.) Trotter*] has never been studied before. In this study, we investigated the effect of Si application on tef plant performance. Plants were grown in soil with or without exogenous application of Na_2_SiO_3_ (0, 1.0, 2.0, 3.0, 4.0, and 5.0 mM), and biomass and grain yield, mineral content, chlorophyll content, plant height, and expression patterns of putative Si transporter genes were studied. Silicon application significantly increased grain yield (100%) at 3.0 mM Si, and aboveground biomass yield by 45% at 5.0 mM Si, while it had no effect on plant height. The observed increase in grain yield appears to be due to enhanced stress resilience and increased total chlorophyll content. Increasing the level of Si increased shoot Si and Na content while it significantly decreased the content of other minerals including K, Ca, Mg, P, S, Fe, and Mn in the shoot, which is likely due to the use of Na containing Si amendment. A slight decrease in grain Ca, P, S, and Mn was also observed with increasing Si treatment. The increase in Si content with increasing Si levels prompted us to analyze the expression of Si transporter genes. The tef genome contains seven putative Si transporters which showed high homology with influx and efflux Lsi transporters reported in various plant species including rice. The tef *Lsi* homologs were deferentially expressed between tissues (roots, leaves, nodes, and inflorescences) and in response to Si, suggesting that they may play a role in Si uptake and/or translocation. Taken together, these results show that Si application improves stress resilience and yield and regulates the expression of putative Si transporter genes. However, further study is needed to determine the physiological function of the putative Si transporters, and to study the effect of field application of Si on tef productivity.

## Introduction

Silicon (Si) is the second abundant element in the earth crust (28%) after oxygen. It has been considered one of the most important elements for some crop plants including rice (Liang et al., 2015a). Si has been shown to improve plant growth, biomass, seed yield and quality, photosynthesis, and resistance to biotic and abiotic stresses (Richmond and Sussman, 2003; Ma, 2004; Cooke and Leishman, 2011; de Oliveira et al., 2016; Zargar et al., 2019). The beneficial roles of Si for plants include increasing plant tissue strength and rigidity, changing of element accumulation pattern, and metabolism of some nutrients (Marxen et al., 2015; Zargar et al., 2019). Si has been shown to enhance the resistance of plant to diseases and pests through formation of physical barriers on the tissue surface (Kim et al., 2002; Silva et al., 2010; Frew et al., 2017). It has also been shown to improve abiotic stress tolerance such as extreme temperature, drought, salinity, and metal toxicity (Zargar et al., 2019). Moreover, Si has been shown to enhance physiological processes such as photosynthesis, respiration, translocation, ion uptake, transpiration rate, root hydraulic conductance, stomatal behavior and conductance, seed germination, mineral nutrition, and plant water relation (Luyckx et al., 2017; Zargar et al., 2019).

Studies on Si uptake, transportation, and accumulation in rice have improved our understanding of the role of Si in plants and its application in agriculture. Plants generally take up Si as silicic acid or monosilicic acid (Si(OH)_4_ or H_4_SiO_4_) (Ma et al., 2001), not in the form of silicon dioxide (SiO_2_), which is the major form of Si in the soil. Plant-available Si in the soil ranges from 10 ppm to over 100 ppm (Liang et al., 2015a), and its concentration in plant leaves varies from 0.1 to 10% on dry weight basis (Ma et al., 2001). Si transport at the lateral roots can be characterized as active, passive, and rejective transports (Raven, 2001; Cornelis et al., 2011). Among those three types of Si transports, passive transport is mediated by membrane transporters belonging to Nod26 (nodulin 26)-like intrinsic proteins (NIPs), a class of the aquaporin family. The first isolated and characterized member of this family that is involved in Si transportation is OsLsi1, which has been shown to mediate Si influx in rice roots (Ma et al., 2006). After Si is taken up by OsLSi1 to the root symplast, an efflux transporter OsLsi2, which is an active Si transporter, facilitates Si release into the xylem from where the Si is translocated from roots to the shoots via transpiration stream (Ma et al., 2007a; Yamaji and Ma, 2011). Both OsLsi1 and OsLsi2 are localized in the plasma membranes of the exo- and endodermal root cells, but OsLsi1 is expressed at the distal side, and OsLsi2 is expressed at the proximal side (Yamaji and Ma, 2007, 2011). Unloading of Si from xylem into xylem parenchyma cells is mostly facilitated by OsLsi6, which is expressed widely in root tips, leaf sheaths, and blades and is localized on the adaxial side of xylem parenchyma cells in the leaf sheaths and leaf blades in rice (Yamaji et al., 2008; Ma et al., 2011). The efflux Si-transporter OsLsi3 is expressed in the first node of rice plant, indicating that Si is reloaded to the vascular bundles and transported to the panicle (Yamaji et al., 2011). Similarly, Si transport mechanism has been studied in other monocot and dicot species including barley (Chiba et al., 2009), maize (Mitani et al., 2009a, b; Xie et al., 2015), sorghum (Markovich et al., 2019), pumpkin (Mitani et al., 2011), tomato (Sun et al., 2020), and soybean (Deshmukh et al., 2013).

The beneficial role of Si in enhancing abiotic stress tolerance and productivity has been studied for crops such as rice and sugar cane (Liang et al., 2015b; de Oliveira et al., 2016; Agostinho et al., 2017; Pardo et al., 2019). In rice, Si treatment has been reported to strengthen the stem by increasing silica deposition in the shoot, increasing the thickness of the culm wall and vascular bundle, enhancing stem stability (Fallah, 2012), forming a physical barrier, and delaying pathogen colonization (Cai et al., 2008; de Oliveira et al., 2016; Wang et al., 2017). Rice and sugarcane are significant Si accumulators among *Poaceae* and have been reported to remove 470 and 700 kg of Si per ha on Si-rich soils, respectively (Pardo et al., 2019), which increased the yield of these crops by up to 50% (Alvarez et al., 2004; Liang et al., 2015b). However, the effect of Si application in low-yielding and lodging-susceptible crops such as tef has never been studied.

Tef [*Eragrostis tef (Zucc.) Trotter*] is a staple food crop for over 60 million people in the Horn of Africa, mainly in Ethiopia, where it is planted on over 3 million hectares annually (Assefa et al., 2015; CSA, 2017; VanBuren et al., 2019). Tef is becoming popular in the Western world due to its nutritional quality and health benefits. The levels of protein, fiber, fat, starch, and vitamin C in tef grains are either superior or similar to other major cereal crops, such as wheat, barley, rice, maize, oat, sorghum (Gebremariam et al., 2014; Daba, 2017; Abewa et al., 2019). Tef grains have low glycemic index which makes them suitable for people with Type 2 diabetes. Moreover, the grains are gluten-free which makes them an alternative diet for people with celiac disease, an immune reaction to gluten-containing diets, which affects about 1 percent of the world population (Spaenij-Dekking et al., 2005; Bergamo et al., 2011; Gebremariam et al., 2014; Shumoy et al., 2018). Tef grains also have higher levels of macro and micronutrients including iron (Fe), calcium (Ca), and copper (Cu) (Abebe et al., 2007; Cheng et al., 2017; Daba, 2017). Most of the nutrients in the tef are considered bioavailable for humans, especially children (Daba, 2017).

Tef is a C4 plant that adapts to a wide ecological zone in Ethiopia, including arid and semi-arid areas prone to drought, and heat where maize, wheat, and rice do not thrive (Cheng et al., 2017). However, tef yield still remains far below its potential with a national average grain yield of only 1.7 ton ha^–1^ in Ethiopia as compared to maize (4 ton ha^–1^) and wheat (2.7 ton ha^–1^) (Cochrane and Bekele, 2018). Tef productivity is constrained by lodging (permanent displacement of the stem from the upright position), diseases, prolonged drought, use of landraces, and cultivars lacking desirable agronomic traits (Ketema, 1997; Assefa et al., 2015; Cannarozzi et al., 2018). Among abiotic stresses, lodging is the most important problem in tef production that causes significant yield losses (Assefa et al., 2011). Some progress has been made to improve lodging tolerance by developing semi-dwarf varieties via mutation breeding (Jöst et al., 2015; Desta et al., 2017; Jifar et al., 2017). Moreover, exogenous application of paclobutrazol (PBZ, an inhibitor of the GA biosynthesis) has been shown to reduce plant height, increase lodging tolerance, and consequently lead to higher shoot biomass and grain yield (Gebre et al., 2012; Plaza-Wüthrich et al., 2016). Agronomic practices including optimization of nitrogen fertilization, plant density, and pest management that can improve crop stability and mechanical resistance are all known to reduce lodging (Wu and Ma, 2016). However, because tef has an inherently weak stem, modification of the stem chemical composition via cellulose, lignin, structural carbohydrate, and silica composition may be needed to increase resistance to lodging, disease, and pests.

In this study, we conducted greenhouse experiments to investigate the effect of Si application on tef growth and productivity. Here we report that Si application increased Si content in the biomass, chlorophyll content, biomass, grain yield, and expression levels of some putative Si transporter genes in leaves but decreased the expression levels of the Si transporter genes in roots. To our knowledge, this is the first report on the beneficial effect of Si in tef.

## Materials and Methods

### Plant Growth and Si Treatment

Ivory tef seeds purchased from Shiloh Farms were used in this study. Ten seeds were germinated in two rows in 10-cm pots containing 350 g soil mix. The soil mix contained one-part Tru soil (Hummert International, Saint Louis, MO, United States), three-part peat moss (Hummert International), two-part multipurpose sand, and three-part medium grade vermiculite (Griffin Greenhouse Supplies, Richmond, VA, United States), which were thoroughly mixed by hand. The soil was fertilized with Scotts Osmocote plus (15–9–12 NPK slow-release fertilizer) at a rate of 0.32 g per pot. The pots were irrigated and arranged on a flat and covered with a plastic dome. Five days after planting, the seedlings were irrigated with 50 mL of 0, 1, 2, 3, 4, and 5 mM Na_2_SiO_3_ every four days. After two weeks, the number of seedlings per pot was reduced to four. After one month of growth, 500 mL of 4.74 g/L Miracle-grow fertilizer solution (24–8–16) was applied per flat containing five pots of each Si treatment. One-month-old plants were also irrigated with 1 L of Si solution corresponding to each treatment directly applied onto the basin holding the pots, and plants were irrigated with DI water between weakly Si treatments. After six weeks, plants were treated with 250 mg/L soluble micronutrient mix M.O.S.T. (J. R. Peter INC, Allentown, PA; pH value) applied per flat. Plants were grown during the summer for four months from April to August in a greenhouse under natural light. Prior to harvesting, plant height measurement was taken, plants were harvested, and the biomass was air-dried to separate the grains. The straw was oven dried at 65°C overnight, and the dry weight was determined.

### Total Elemental Analyses

Plant materials (seed and biomass) were oven-dried at 65°C overnight and finely ground using a Waring Laboratory blender. Elemental analysis was performed according to Pardo et al. (2019). Briefly, to 200 mg of ground material 7 mL of trace-metal grade nitric acid was added. The biomass was then digested using Mars 6 microwave digester (CEM Corporation, Matthews, United States) at 200°C for 10 min. The resulting solution was transferred to a 50-mL falcon tube and diluted to 35 g with deionized water and centrifuged at 500 g for 5 min to separate the Si-rich precipitate from the acid fraction. The acid fraction was decanted into another conical tube and was diluted to 2% acid for analysis by using Inductively Coupled Plasma Optical Emission Spectroscopy (ICP-OES, Thermo Fisher Scientific, Waltham, United States) for elements Al, B, Ca, Cu, Fe, K, Mg, Mn, Na, P, S, and Zn. The Si-rich precipitate that settled at the bottom of the conical tubes was rinsed with DI H_2_O three times and centrifuged, and the supernatant was decanted. After the final rinse, 15 mL of 2 M NaOH was added to the precipitate, which was allowed to stand for three days at room temperature to dissolve the Si precipitate. The Si content was then analyzed colorimetrically by the molybdenum blue technique according to Kraska and Breitenbeck (2010) using an Evolution 60S UV-visible spectrophotometer (Thermo Fisher Scientific) at 630 nm. A standard reference material WEPAL 883 carnation straw was used to assess the recovery of other elements and was included in each set of digestions along with a blank sample without the plant material. All concentrations were calculated on a dry-weight basis.

### Analysis of Putative Si Transporter Genes

The sequence of seven putative Si transporter transcripts was obtained from the recently released tef draft genome sequence (Pardo et al., 2019). Analysis of tef putative Si transporter proteins’ physiochemical properties including number of amino acids, molecular weight, theoretical isoelectric point, and instability index was performed using the Protparam tool available in ExPASy59^1^. Protein secondary structure was analyzed using the Sopma tool available in Prabi^2^. The number of helices was analyzed by Phyre2^3^, and transmembrane–helix prediction was carried out using TMHMM^4^ and Protter^5^ tools. The conserved motifs of tef putative silicon transporters were predicted using the MEME tool^6^ with default parameters. The phylogenetic tree of 38 Si transporter proteins in tef and other monocots (Supplementary Table 1) was constructed using MEGA X (version 10.1.8) software tool (Kumar et al., 2018). Protein sequences were aligned using Clustal X2 (Thompson et al., 1997) and subjected to construct phylogenetic tree using Neighbor-Joining method with 1000 bootstrap iterations, with 60% cutoff value.

### RNA Isolation and Quantitative PCR

Plants were grown in a controlled growth chamber under hydroponic conditions containing 1/4 strength modified Hoagland’s solution (Epstein and Bloom, 2005) with or without 3 mM Na_2_SiO_3_ (pH = 5.8) for one month. Total RNA was extracted using GeneJET RNA Purification Kit (Thermo Fisher Scientific) from root tip (∼5 cm from the tips), nodes, leaves, and inflorescence of two-month old plants that were ground under liquid nitrogen. The RNA samples were treated with DNase I (Thermo Fisher Scientific) to eliminate contaminating genomic DNA. First-strand cDNA was generated using High-Capacity cDNA Reverse Transcription Kit (Applied Biosystems) according to the manufacturer’s protocol. Quantitative real-time PCR was performed using PowerUp^TM^ SYBR^®^ Green pre-formulated 2 × Master Mix (Applied Biosystems). The relative transcript abundance of putative *EtLsi1* and *EtLsi2-1*, *EtLsi2-2*, *EtLsi2-3* and *EtLsi2-4*, and *EtLsi6* were measured in roots, shoot, and inflorescence in control and Si-treated plants. Each qPCR reaction contained 1 μL of cDNA, SYBR green SuperMix, and forward and reverse primers. Gene-specific sense and antisense primers (Supplementary Table 2) were used for amplification, and relative expression of the tef Lsi genes was quantified using RNA polymerase II as a housekeeping gene after validating its stability in control and Si treated samples. The qPCR parameters were as follows: 95°C for 30 s, 30 cycles of 95°C for 15 s, 62°C for 30 s, 68°C for 45 s, and a final incubation at 72°C for 5 min followed by melting curve analysis. Relative expression level was calculated using the ΔΔCT method (Livak and Schmittgen, 2001) available on QuantStudio 3 software (Thermo Fisher Scientific). Gene expression in the untreated control leaves was used as a calibrator to determine differential expression of the *Lsi* genes in response to Si and in different tissues.

### Total Chlorophyll Content

The total chlorophyll content of control and Si-treated leaf tissues was determined as described by Whitham et al. (1986). Samples were obtained from fully expanded top leaf of two-month-old soil grown plants. To extract chlorophyll pigments, 1 g of leaf tissue was homogenized with 80% acetone solution. The leaf extracts were filtered using Whatman filter paper, and the volume was adjusted to 100 mL using 80% acetone. The optical density of the chlorophyll extract was read at 645 and 663 nm using an Evolution 60S UV-visible (Thermo Fisher Scientific). Total chlorophyll content of the leaf tissue extract was calculated based on the following formula:

$$\begin{array}{c} \mathrm{Total}\mathrm{chlorophyll}\left(\text{mg}/\text{g}\right)=20.2\left(\text{OD}645\right)+\\ \;8.02\left(OD663\right)\times \frac{volume\left(ml\right)}{\left(1000\times weight\left(g\right)\right)} \end{array}$$

where OD = optical density reading of the chlorophyll extract at the specific wavelength; volume = final volume of the 80% acetone-chlorophyll extract (100 mL), and weight = fresh weight in grams of the tissue extract.

### Data Analysis

Treatments were replicated at least four times, and two independent experiments were conducted. Data were analyzed by one-way ANOVA using the PROC GLM procedure (Westfall et al., 1996). After the significant *F*-tests, the Tukey multiple comparison was used to separate the means (*P* < 0.01).

## Results

### Si Treatment Improves Agronomic Traits

**FIGURE 1:**
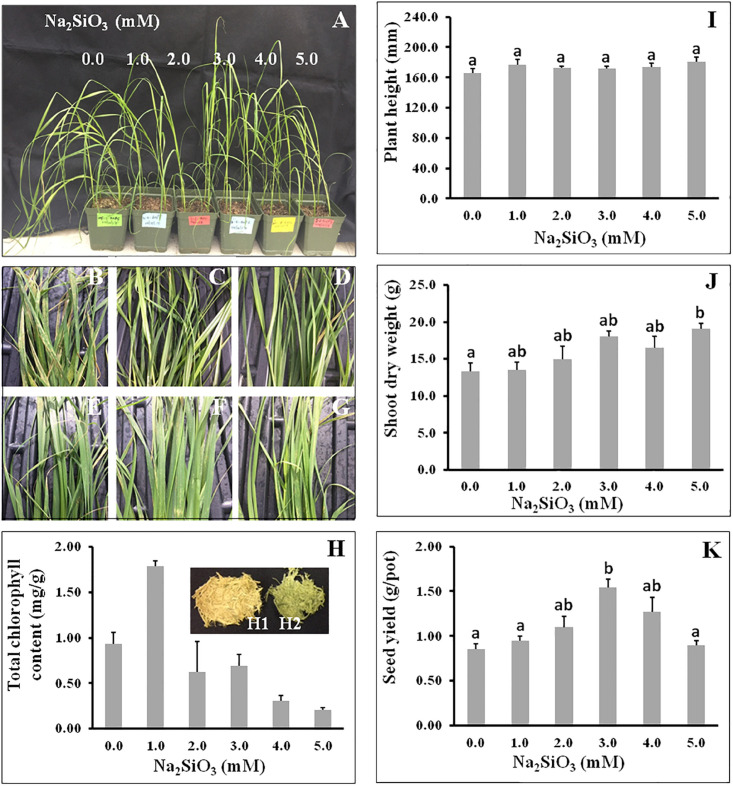
The response of tef to varying Si treatments. **(A)** One-month-old control (0 mM) and Si-treated (1.0, 2.0, 3.0, 4.0, and 5.0 mM Na_2_SiO_3_) plants. **(B–G)** Decreasing leaf injuries with increasing Si treatment from 0 to 5 mM Na_2_SiO_3_. **(H)** Total chlorophyll content of leaves as in panels **(B–G)**. Note that at high Si concentration, extraction of chlorophyll with 80% acetone is not complete (*Inset* H2); pictures were taken after chlorophyll extraction. **(I)** Plant height at maturity, **(J)** shoot dry weight, and **(K)** seed yield in response to Si treatment. N, is not affected by Si treatment. Bars represent means ± SE, *n* = *5*). Bars bearing the same letter are not significantly different.

Tef seedlings were treated with varying concentrations of Si (0, 1.0, 2.0, 3.0, 4.0, and 5.0 mM), and parameters including plant height, total chlorophyll, and shoot and root dry weight were determined. As shown in Figure 1A, the appearance of one-month-old control and lower Si concentration was slightly different from plants treated with higher Si levels. Si treatment appeared to improve plant architecture. Plants treated at higher Si levels developed leaves that appeared erect as compared to the control with larger leaf angle, in which the leaves appeared like an arc. A similar effect of Si on plant architecture has been reported in rice (de Oliveira et al., 2016). Si treatment had no significant effect on plant height measured at maturity (Figure 1I).

Our findings showed that control plants experienced blight-like symptoms (Figure 1B), primarily on the leaves and leaf sheath as compared to plants treated with Si, and the leaves of Si-treated plants looked healthier and greener (Figures 1C–G). This result suggests that Si may confer disease tolerance in tef; however, further study in ongoing to identify the pathogen and the effect of Si in disease tolerance in detail.

The total chlorophyll in leaves increased from control to the lowest Si treatment, but then decreased with increasing Si (Figure 1H). The chlorophyll content at 1.0 mM was nearly twice that of the control without Si addition. However, the amount of chlorophyll extracted from the leaves decreased with increasing level of Si. This decrease in chlorophyll content may be due to increased Si deposition in the tissues, which appeared to inhibit the release of chlorophyll pigment from the leaf tissues during extraction. As shown in the Figure 1H *Inset* H1 and H2, after extraction with acetone, the color of the shoots at 4 mM Si (Figure 1H *Inlet* H2) remained greener than that of control plants (Figure 1H *Inlet* H1).

In this study, the effect of Si on biomass and grain yield was also determined. Shoot biomass increased with increasing Si treatment (Figure 1J). At 5 mM Si, shoot biomass yield increased by approximately 45% as compared to the control. An increase in grain yield was also observed with increasing Si application up to 4 mM. Maximum grain yield was measured at 3.0 mM Si. The grain yield at 3 mM Si was nearly twice that of the control. However, grain yield decreased significantly with increasing Si contents from 3.0 to 5.0 mM (Figure 1K).

### The Effect of Si Treatment on Mineral Nutrients

**FIGURE 2:**
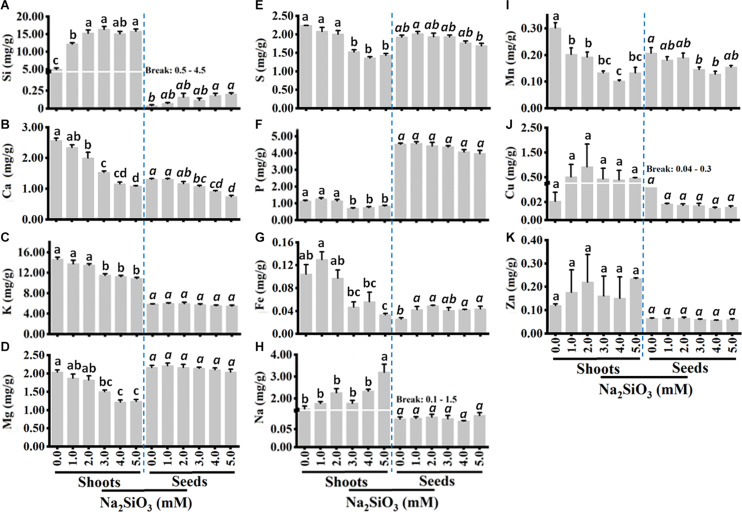
Tef shoot and seed mineral contents of Si **(A)**, Ca **(B)**, K **(C)**, Mg **(D)**, S **(E)**, P **(F)**, Fe **(G)**, Na **(H)**, Mn **(I)**, Cu **(J)**, and Zn **(K)**. Plants were grown without or with varying Si treatments as in panel. Shoot biomass was dried, and mineral content was determined as described in the section “Materials and Methods.” Bars represent means ± SE (*n = 4*). Bars bearing same letter are not significantly different.

To determine the effect of Si application on mineral nutrient accumulation, shoot and grain were analyzed using ICP-OES. The results show that shoot Si content increased with increasing Si treatment up to 3 mM. At 3 mM Si, shoot Si content was three-fold higher than the control. Similarly, seed Si content also increased with Si application. There was no increase in Si content after 3, and 2 mM in shoots and seeds, respectively (Figure 2A). The contents of Si in the shoots were up to 100-fold higher than seed Si content suggesting that the majority of Si remains in the stem and leaves.

Si application has a dramatic effect on macro and micronutrient accumulation in the shoots and slightly affected Si content in the grains. In the shoot, the content of nutrients including Ca, K, Mg, S, P, and Mn decreased significantly (*P* < 0.05) with increasing Si levels (Figures 2B–F,I). At 5 mM Si, shoot Ca decreased by nearly three-fold as compared to the control (Figure 2B), while shoot K, Mg, S, and P decreased by 25, 50, 60, and 30%, respectively (Figures 2C–F,I). Si also slightly decreased seed Ca, S, and P, whereas seed K, Na, Cu, Zn, and Mg were not affected by Si treatment (Figure 2). On the contrary, the concentration of Na increased significantly at 5 mM (Figure 2H) while shoot Fe, Cu, and Zn increased slightly by Si treatment (Figures 2G,J,K) up to 2 mM. There was no marked effect of Si on seed micronutrient content except Mn, which significantly decreased with increasing Si treatment compared to the control without Si (Figure 2I). Taken together, Si application decreases the content of most macronutrients in the shoot but increased the content of Si and Na. However, the effect of Si contest of most nutrients was negligible in the grains except Ca content which decreased significantly with Si, and grain Fe content which showed a significant increase with Si treatment.

### Identification and Structural and Phylogenetic Analysis of Putative Si Transporters

The role of Si transporters in mediating Si uptake and translocation in various plant species has been studied. However, Si transport in tef and transporters that potentially mediate Si efflux remained unknown. As shown in Figure 2A, Si treatment increased shoot and seed Si content, suggesting the presence of membrane transporters that facilitate Si uptake and translocation. We searched homologs of known Si transporters in tef draft genome database (Pardo et al., 2019) and identified seven putative Si transporters based on homology to rice and barley Si efflux and influx transporters. The tef contiguous sequences include *EtLsi1-1* (ID: Et_1B_012646), *EtLsi1-2* (Et_1B_012509), *EtLsi2-1* (Et_4A_034104), *EtLsi2-2* (Et_4B_037456), *EtLsi2-3* (Et_1B_012902), *EtLsi2-4* (Et_4A_035418), and *EtLsi6-1* (Et_2A_017757). The sequence information of the tef transporters is provided in Supplementary Table 1.

**FIGURE 3:**
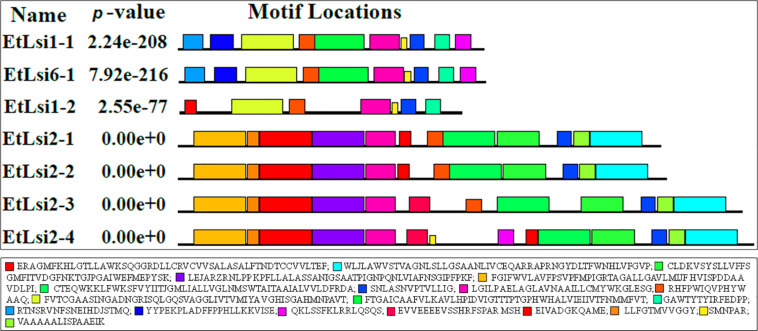
Conserved motifs of tef putative silicon transporter proteins were predicted by MEME tools. 20 colors indicated different sequences and motifs identified.

To analyze the structure of tef putative Lsi proteins, the nucleotide sequences were translated to amino acid sequences using BioEdit version 7.2.5^7^. The number of amino acid residues varied from 273 in (EtLsi1-2) to 556 in (EtLsi2-4), with corresponding molecular weights of 28.91 and 60.39 kDA, respectively (Table 1). The lowest and highest theoretical iso-electric points (pI) of 5.57 and 7.08 were predicted for EtLsi2-4 and EtLsi6-1 proteins, respectively. Variation in secondary structures of the Si transporter proteins was also observed. The percentage of alpha helices, beta sheets, beta turns, and random coil ranged from 34.12 to 50.00, 13.67 to 19.93, 4.05 to 6.97, and 28.81 to 41.89, respectively (Table 1). Except for EtLsi2-4, all the proteins possess the NPA or SPA motifs, which are major features of the major intrinsic protein superfamily. The SPA domains were identified in the EtLsi2-type proteins, while the NPA motif was detected in EtLsi1 and EtLsi6 proteins (Table 1). A total of 10 conserved motifs were deduced in EtLsi1-1 and EtLsi6-1 transporters, while EtLsi1-2 only had 7 conserved motifs as determined using the MEME tool (Figure 3), whereas 12 conserved motifs were identified in EtLsi2-1, EtLsi2-2, and EtLsi2-3, and 14 in EtLsi2-4 (Figure 3). The predicted 3-D structure of all tef Lsi transporter proteins showed that the proteins form a pore structure with helices, which is a typical characteristics characteristic of a transporter/channel proteins. Among seven Si transporter proteins, the number of alpha helices ranged from 11 to 23. Membrane topology of the tef Lsi proteins was predicted using the TMHMM and Protter tools. All the deduced Si transporter proteins contained 5–11 transmembrane helices, suggesting that all the proteins are membrane binding and may play a role in Si transport across cellular membranes (Table 1).

**TABLE 1:** Characteristics of putative silicon-transporter proteins identified in tef.

Gene name	Gene ID	Physiochemical properties of protein						Secondary structure (%)				Number of helices	TMHMM (TMHs)	Protter (TMHs)
		aa length	MW (kDa)	pI	NAP/SPA domain	Instability index	Stable or unstable	Alpha helix	Beta sheet	Beta turns	Random coil			
EtLsi1-1	Et_1B_012646	296	31.73	6.74	NPA	27.86	Stable	34.12	19.93	4.05	41.89	11	6	6
EtLsi1-2	Et_1B_012509	273	28.91	8,82	NPA	29.00	Stable	35.53	19.78	4.76	39.93	11	5	7
EtLsi2-1	Et_4A_034104	466	49.69	8.36	SPA	35.28	Stable	49.79	16.09	5.15	28.97	19	11	11
EtLsi2-2	Et_4B_037456	472	50.26	8.84	SPA	34.75	Stable	50.00	16.10	5.08	28.81	17	10	10
EtLsi2-3	Et_1B_012902	545	58.33	6.16	SPA	31.98	Stable	44.22	17.06	6.97	31.74	23	10	11
EtLsi2-4	Et_4A_035418	556	60.39	5.57	-	39.75	Stable	45.14	13.67	6.29	36.69	22	9	10
EtLsi6-1	Et_2A_017757	296	31.73	7.08	NPA	40.85	Unstable	37.50	17.23	5.74	34.89	11	6	6

*aa, amino acid; MW, molecular weight; pI, isoelectric point; kDa, kilo dalton; TMHs, transmembrane helices.*

**FIGURE 4:**
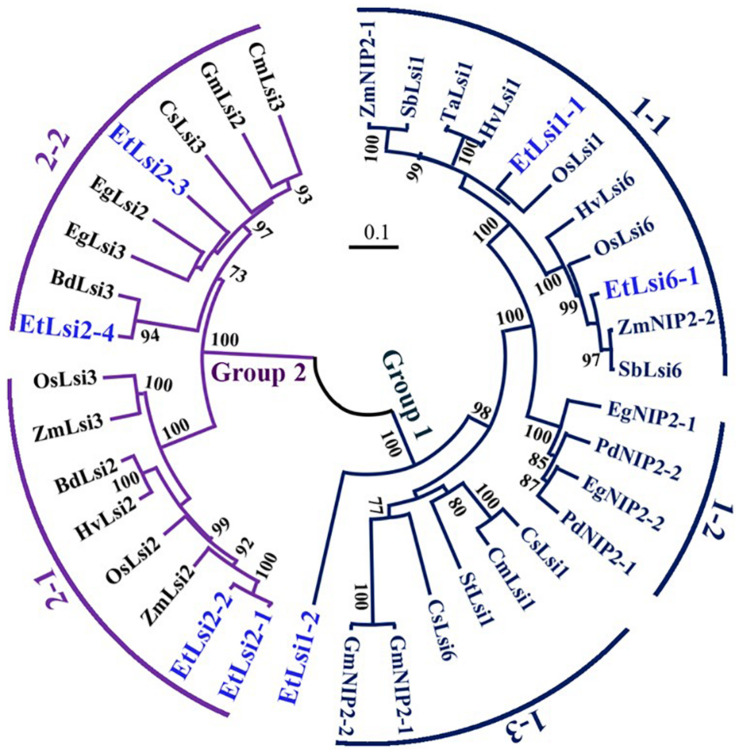
Phylogenetic tree of the Si transporter proteins family. The phylogenetic tree was generated using MEGA X software. Protein sequences were aligned using Clustal X2, and amino acid sequences are presented in Supplemental Table 1. The scale bar represents the evolutionary distance, expressed as the number of substitutions per amino acid. The following is accession numbers of registered genes and tef Lsi-like genes. Protein name starts with the first letter of genus and species names (Bd, *Brachypodium distachyon*; Cm, *Cucurbita moschata*; Cs, *Cucumis sativus*; Eg, *Elaeis guineensis*; Et, *Eragrostis tef*; Gm, *Glycine max*; Hv, *Hordeum vulgare*; Os, *Oryza sativa*; Pd, *Phoenix dactylifera*; Sb, *Sorghum bicolor*; St, Solanum tuberosum; Ta, *Triticum aestivum*; Zm, *Zea mays*).

Most of the tef transporters showed high similarity with rice and maize homologs while EtLsi2-3 showed higher homology with oil palm (EgLsi2). As shown in the phylogenetic tree, 38 Lsi protein families are clustered into two major groups (Figure 4); Group 1 encompasses the NIP proteins, including Lsi1 and Lsi6, generally termed as Si influx transporters, and Group 2 encompasses the Si efflux transporters, including Lsi2 and Lsi3 which belong to belong to anion transporters family (Ma et al., 2007a; Jadhao et al., 2020). Group 1 consists of three subgroups, Group 1-1, which includes transporters from the Poaceae family, Group 1-2 which includes transporters from the *Arecaceae* family, and group 1-3 which includes transporters from dicotyledonous plants. EtLsi1-1 is closely related to OsLsi1 (Ma et al., 2006), HvLsi1 (Chiba et al., 2009; Yamaji et al., 2012), and TaLsi1 (Montpetit et al., 2012), but EtLsi1-2 is distantly related to the *Poaceae* Lsi transporters in Group 1. EtLsi6-1 showed high similarity with ZmNIP2-2 (Mitani et al., 2009b), SbLsi6 (Markovich et al., 2019), OsLsi6 (Yamaji et al., 2008; Ma et al., 2011), and HvLsi6 (Yamaji et al., 2012). EtLsi1-1 and EtLsi1-6 belong to group 1–1. Two subgroups (subgroup 2-1 and 2-2) are identified in Group 2. EtLsi2-1 and EtLsi2-2 are in group 2-1, and showed higher similar with ZmLsi2 (Mitani et al., 2009a) and OsLsi2 (Ma et al., 2007a; Yamaji and Ma, 2011). EtLsi2-3 and EtLsi2-4 and EtLsi2-3 showed high similarity with EgLsi2 (NCBI ID: XP_019710862.1) and EgLsi3 (NCBI ID: XP_019705523.1), and EtLsi2-4 is closely related to brachypodium BdLsi3 (NCBI ID: XP_014752591.1).

### Spatial Expression of Si Transporters

**FIGURE 5:**
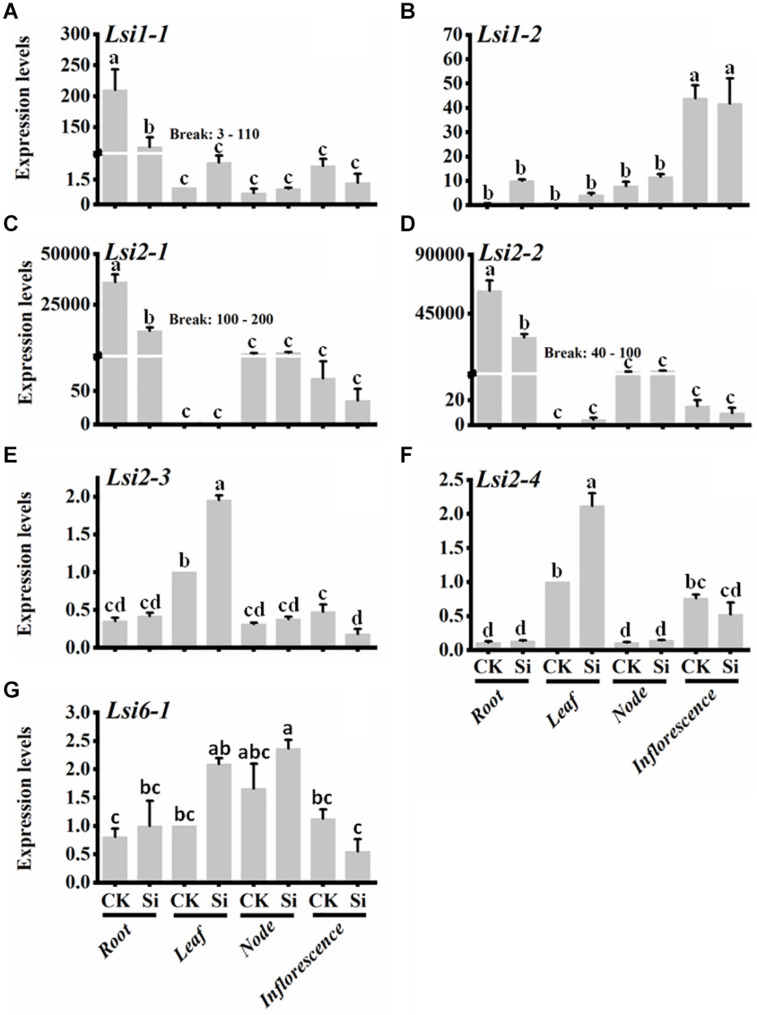
Expression pattern of Si-transporter genes in different tissues grown without or with 3 mM Na_2_SiO_3_. **(A)** *Lsi1-1*, **(B)** *Lsi1-2*, **(C)** *Lsi2-1*, **(D)** *Lsi2-2*, **(E)** *Lsi2-3*, **(F)** *Lsi2-4*, and **(G)** *Lsi6-1*. Bars represent mean fold-change compared control for each tissue ± SE (*n = 4*). Bars bearing same letter are not significantly different.

**FIGURE 6:**
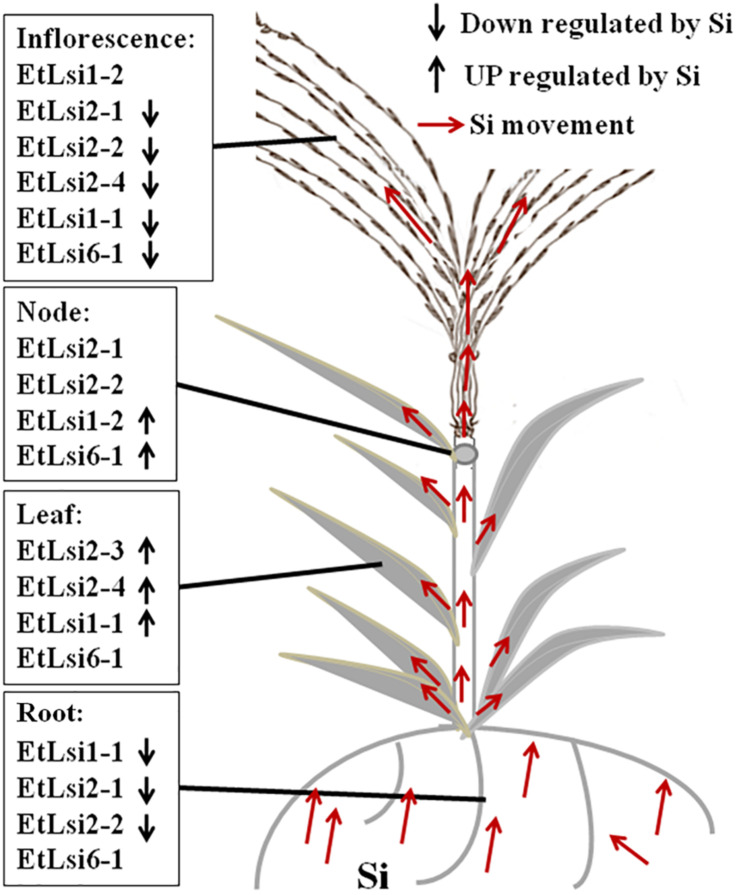
A simple model showing possible localization of the putative tef Si transporters. The model is based on differential expression by Si and in different tissues as shown in Figure 5.

To analyze the expression of Si transporters in tef, plants were grown in hydroponic solution containing 0 or 3 mM Si, and gene expression was analyzed by qPCR. As shown in Figure 5, expression of the seven Si-transporter genes was spatially regulated in the four organs (root, leaf, note, and inflorescence) analyzed. Moreover, gene expression was regulated by Si treatment (Figure 5). The expression of *EtLsi1-1* (Figure 5A), *EtLsi2-1* (Figure 5C), and *EtLsi2-2* (Figure 5D) in roots was significantly higher (*p* < 0.05) than the shoot tissues (node and inflorescence, while the expression of *EtLsi2-3* (Figure 4E), *EtLsi2-4* (Figure 5F), and *EtLsi6-1* (Figure 5G) was significantly higher in the leaf (*p* < 0.05). The expression of *EtLsi1-2* (Figure 5B) was significantly higher in the inflorescence (*p* < *0.05*). The expression of *EtLsi1-1*, *EtLsi2-1*, and *EtLsi2-2* in the root was decreased significantly by Si (*p* < *0.05*) while *EtLsi1-2*, *EtLsi2-3*, *EtLsi2-4*, and *EtLsi6-1* were slightly increased by Si treatment in some tissues, suggesting their roles in Si transport. The expression of *EtLsi2-1*, *EtLsi2-2*, and *EtLsi1-2* is either undetected or extremely low in the leaf. Expression of *EtLsi2-1* and *EtLsi2-2* was detected in the nodes but at a lower level than the roots (*p* < *0.05*). Expression of *EtLsi1-1*, *EtLsi2-1*, *EtLsi2-3*, and *EtLsi6-1* in the inflorescence was slightly reduced by Si. In addition, transcripts of *EtLsi1-1*, *EtLsi1-2*, *EtLsi2-1*, and *EtLsi2-2* were more abundant than those of all the other transcripts in the roots and shoots (Figure 5), indicating that these four genes may play a major role in Si absorption and transportation. The expression of tef Si transporter gene in response to Si treatment is summarized in Figure 6. The expression of *EtLsi1-1*, *EtLsi2-1*, and *EtLsi2-2* is downregulated by Si in the roots while the expression of *Lsi2-3* and *EtLsi2-4* is upregulated by Si in the leaves. None of the *Lsi* genes was significantly upregulated by Si in the nodes. Of the seven *EtLsi* genes, only *Lsi*2-3 was significantly downregulated by Si in the inflorescence (*p* < *0.05*).

## Discussion

Silicon has been shown to improve agronomic traits in various crops, but it remained unknown whether Si application could improve the performance of low yielding “orphan” crops including tef. In this study, we evaluated the response of tef to varying Si levels and determined several agronomic parameters including biomass and grain yield, plant height, chlorophyll content, and the expression of putative Si transporter genes.

### Si Improves Biomass and Grain Yield in Tef

Si application has been shown to improve biomass, grain yield, and tiller number in several crops (Deren et al., 1994; Pati et al., 2016). Similarly, long-term field trials with slow-released potassium silicate have been shown to increase wheat yield by 13.8% on average, and by up to 50% in rice and sugarcane (Alvarez et al., 2004; Liang et al., 2015a). In the current study, the grain yield of tef was increased by about 100% in greenhouse-grown plants treated with 3.0 mM Si as compared to the control without Si (Figure 1K), while shoot biomass increased by 35% at Si 3.0 mM Si and by 45% at 5 mM Si (Figure 1H). These findings reveal the potential of Si application in improving tef productivity. Given the current low grain yield in tef (1.7 ton ha^–1^) even in its country of origin Ethiopia (CSA, 2017), Si application may significantly boost tef productivity. However, the findings need to be validated under field conditions. In this study, Si was applied in the form of Na_2_SiO_3_; however, for large-scale field application other Si fertilizers such as calcium silicate, calcium magnesium silicate, and potassium silicate etc. need to be tested because Na_2_SiO_3_ may lead to field salinization.

In rice, silicon treatment has been shown to strengthen the stem by increasing silica deposition and the thickness of the culm wall and vascular bundle and enhance stem stability and lodging tolerance (Fallah, 2012). Lodging is the most critical constraint in tef production (Assefa et al., 2011). In this study, Si did not appear to have improved lodging tolerance. Plants were grown in small pots, and although Si-treated plants grew upright with erect leaves prior to anthesis (Figure 1A), lodging was observed during grain filling in all plants grown with or without Si. The observed increase in grain and biomass yield is likely attributed to high chlorophyll content and stress tolerance as observed in Figures 1B–G. Leaves of Si-treated plants looked healthier and had less blight-like symptoms (Figures 1C–G), with high total chlorophyll content (Figure 1J), which might have enhanced photosynthesis; however, further study is needed to understand the beneficial role of Si in yield improvement in tef. Si applications in rice have been shown to improve biotic stress tolerance including diseases such as blast (Rodrigues et al., 2003a; Sun et al., 2010), sheath blight (Rodrigues et al., 2003b), and brown spot (Ning et al., 2014), as well as insects (Frew et al., 2017).

### Si Treatment Modulates the Accumulation Pattern of Mineral Nutrient in Tef

Exogenous Si application significantly enhances Si and micronutrient (Fe, Mn, and Cu) content while it reduced the macronutrient content. Plant species and even genotypes of the same species vary in Si accumulation (for reviews see Ma et al., 2007b). In shoots, Si content was 0.5% (4.96 mg/g) in the controls which was increased to 1.22% (12.2 mg/g) at 1 mM Si (Figure 2A). Si content in plant tissues varies with Si level and plant species. At 1.4 mM, Si application in rice, 39 mg/g Si in the shoot, and 42.62 mg/g in the leaf have been reported (Lin et al., 2019). Similarly in chickpea (*Cicer arietinum*), 3.0 mg/g Si was reported in the shoots (Broadley et al., 2011), and higher Si content (6.4 to 10.2 mg/g Si) has been reported in shoots of sugarcane (*Saccharum officinarum*) (Deren, 2001). The Si content in tef seeds was much lower than that of straw. In the seeds of control tef plants, 0.04 mg/g was detected which showed a five-fold increase to 0.2 mg/g at 5.0 mM Si treatments (Figure 2A). In barley (*Hordeum vulgare*) grains, Si content ranging from 1.2 to 3.8 mg/g has been reported (Ma et al., 2003).

Studies have shown that Si treatment improves macro (N, P, K, Ca, and Mg) and micronutrient (B, Zn, Fe, Cu, and Mn) accumulation in some plants under different stress conditions including high salt (Zargar et al., 2019), drought (Gunes et al., 2008; Emam et al., 2014), and heavy metals Mn, Cr, Cu, and Cd (Dragišić Maksimović et al., 2012). Our findings contrast with former studies. Si application decreased the content of nutrients including K, S, Ca, Mg, P, and Mn (Figure 2) while it increased the content of micronutrients Fe, Cu, and Zn. This decrease in macro and micronutrient content with increasing Si application could partly be due to a growth dilution because an increase in biomass was observed with increasing Si levels (Figure 1J).

The application of Si has been shown to improve the uptake of P and K in aboveground rice biomass (Pati et al., 2016; Crooks and Prentice, 2017; Cuong et al., 2017). However, Ma and Takahashi (1990) showed that addition of Si was not accompanied by an increase in shoot P concentration. We observed a significant decrease in both shoots and grain Ca with increasing Si levels (Figure 2B), and similar results were observed for shoot K, Mg, S, and P content (Figure 2). However, Jang et al. (2018) showed that Mg level in rice was significantly increased in the presence of Si as compared to the control. An increase in Mg in response to Si has also been reported in poinsettia (Hu et al., 2019). A decrease in Ca content by 11.8% to 15.8% due to Si treatment has been reported in rice (Jang et al., 2018), whereas Si application increased N, P, Ca, Fe, and Mg contents in roots and leaves of tomato (Abdalla, 2011; Li et al., 2015), canola (Farshidi et al., 2012), and cucumber (Khoshgoftarmanesh et al., 2014) under salt stress. On the contrary, Si had no effect on shoot Ca concentration in plants grown under non-saline conditions (Khoshgoftarmanesh et al., 2014).

In this study, the Na content in tef shoots increased with increasing Na_2_SiO_3_ (Figure 2H), but the Na content in the grains was not significantly increased by Na_2_SiO_3_. Si application in hydroponic solution also increased the uptake of macronutrients (Ca, Mg, P, and K) by wheat under Cr (Tripathi et al., 2015), Cu (Keller et al., 2015), and Cd (Rizwan et al., 2012) stresses. The levels of Mn in both shoots and seeds decreased with increasing Si treatment (Figure 2I). Similarly, Si has been reported to alleviate Mn toxicity through decreasing Mn levels in rice, common bean, cowpea, cucumber, and pumpkin (Rogalla and Römheld, 2002; Li et al., 2012; Che et al., 2016; Agostinho et al., 2017). Our data showed that Cu contents in tef shoots increased with increasing Si levels up to 2.0 mM and then decreased with further increasing Si level whereas a slight decrease in seed Cu was observed in the seeds with increasing Si treatment (Figure 2J). In poinsettia, Cu content in shoot was not significantly affected by supplementary Si (Hu et al., 2019). As shown in Figure 2K, Si treatment slightly increased shoot Zn content. A contrasting result was reported in maize and cotton with Si application under Zn toxicity condition (Anwaar et al., 2014; Bokor et al., 2015). The differences in mineral composition between tef and other crops in response to Si may be due the difference in the uptake and distribution mechanisms among the different plant species. Tef accession used in this study may be a poor Si accumulator. There are over 5000 tef accessions in the world, and screening of these germplasm may lead to identification of a high Si accumulator. The source of applied Si is another factor that could affect the availability of minerals. In this study, we used a Na-containing Si compound. Because Na is toxic to plants at high levels (>3 mM), increasing the level of Na_2_SiO_3_ fertilizer might have reduced mineral uptake. Moreover, application of Na_3_SiO_3_ may increase the soil pH above 7.0 at which most nutrients are not available for uptake. Therefore, further study is needed to test the effect of other Si fertilizers (calcium silicate, calcium magnesium silicate, and potassium silicate) and soil pH on mineral accumulation. Plant available Si and its beneficial effect depend on the Si fertilizer used (Seyfferth et al., 2018).

### Expression Patterns of Putative Si Transporter Genes Are Regulated by Si Application

The observed increase in Si accumulation in the shoots and seeds (Figure 2A) indicates that tef possesses Si transporters that are involved in the uptake and translocation of Si. We identified seven putative Si transporters gens and tentatively named them *EtLsi1-1*, *EtLsi1-2*, *EtLsi2-1*, *EtLsi2-2*, *EtLsi2-3*, *EtLsi2-4*, and *EtLsi6-1* (Table 1 and Supplementary Table 1). As shown in Figures 3, 4, these transporters showed homology with previously reported Si transporters in other crop species (Ma et al., 2006, 2007a; Chiba et al., 2009; Mitani et al., 2009a, b; Grégoire et al., 2012; Xie et al., 2015; Vivancos et al., 2016; Markovich et al., 2019). As shown in the phylogenetic tree (Figure 4), homologs of the influx Si transporters EtLsi1-1, EtLsi1-2, and EtLsi6-1 were clustered into Group 1 together, and efflux Si transporters EtLsi2-1, EtLsi2-2, EtLsi2-3, and EtLsi2-4 were clustered into Group 2.

We studied the expression of the tef Si transporter genes using quantitative PCR. The genes were differentially expressed based on plant tissue or Si treatment (Figure 5). The expression patterns of *EtLsi1-1*, *EtLsi2-1*, and *EtLsi2-2* showed that they were primarily expressed in roots and suppressed by addition of Si (Figures 5A,C,D), similar to the rice homologs *OsLsi1* (Ma et al., 2006; Yamaji and Ma, 2007) and *OsLsi2* (Ma et al., 2007a; Yamaji and Ma, 2011); *OsLsi1* and *OsLsi2* play roles as Si-influx and efflux transporters in rice root, respectively. Similarly, the soybean *GmNIP2-1* and *GmNIP2-2* (Deshmukh et al., 2013), cucumber *CsLsi1* and *CsLsi2* (Sun et al., 2017; Sun et al., 2018), wheat *TaLsi1* (Montpetit et al., 2012), and maize *ZmLsi1* and *ZmLsi2* (Bokor et al., 2015) are also primarily expressed in roots. However, the expression of barley *HvLsi1* (Chiba et al., 2009), maize *ZmLsi* (Mitani et al., 2009a), tomato *SlLsi1* and two *SlLsi2-1* genes (Sun et al., 2020), and wheat *TaLsi1* was not affected by exogenous Si application although they were mainly expressed in the roots (Montpetit et al., 2012). Like *OsLsi1* (Ma et al., 2006), *EtLsi1-1* transcripts were also detected in tef leaf, with increasing level of expression in response to Si. The *EtLsi1-1* was also expressed in the inflorescence at lower levels, but its expression was reduced by Si (Figure 5A), suggesting that *EtLsi1-1* may have a role in these organs. Transcripts of *EtLsi2-1* (Figure 5C) and *EtLsi2-2* (Figure 5D) were also discovered in notes and inflorescence, not in leaves, and the expression of both *EtLsi2-1* and *EtLsi2-2* genes was suppressed in the inflorescence, and both genes did not respond to Si treatment in the node, suggesting that *EtLsi2-1* and *EtLsi2-2* have some functions in note and inflorescence.

Interestingly, *EtLsi1-2* is distantly related to *EtLsi1-1*, which is a homolog of *OsLsi1* homolog, Figure 4. *EtLsi1-2* is mainly expressed in the inflorescence and showed no response to Si addition, and it was expressed at very low levels in roots, leaf, and nodes, but the expression levels of *EtLsi1-2* were enhanced by Si in three organs root, leaf, and node (Figure 5B). The pattern of *EtLsi 1-2* expression may suggest that it is likely involved in Si transport into the seeds. *EtLsi2-3* (Figure 5E) and *EtLsi2-4* (Figure 5F) were mainly expressed in the leaf, and their expression is enhanced by Si. The expression of EtLsi2-3 and EtLsi2-4 was lower in roots, nodes, and inflorescence as compared to the leaf. In soybean, *GmNIP2-1* and *GmNIP2-2* genes were also detected in roots and shoots, but their expressions decreased with increasing Si (Deshmukh et al., 2013). The expression of *EtLsi6-1* was higher in leaf and node but was not affected by Si (Figure 5G). The rice *Lsi6* was expressed more in the leaf sheath and leaf blades as well as in the root tips (Yamaji et al., 2008; Yamaji and Ma, 2009). The expression pattern of *EtLsi2-3*, *EtLsi2-4*, and *EtLsi6-1* suggests that they may have roles in transporting Si in tef leaf. Like *EtLsi6-1*, *OsLsi2*, and *OsLsi3* were also highly expressed in the first node (Yamaji et al., 2011), and *OsLsi6* was greatly enhanced in node I below the panicles (Yamaji and Ma, 2009; Yamaji et al., 2012), and knockout of *OsLsi6* has been shown to decrease Si accumulation in the panicle but increase Si level in flag leaf (Yamaji and Ma, 2009). Expression of the *EtLsi6-1*, *EtLsi2-1*, and *EtLsi2-2* may suggest a role in Si transport at the node.

A model of Si transportation in tef plant can be deduced based on the expression analysis of seven putative Si-transporter genes, and other former works in rice, barley, and sorghum. The putative influx Si-transporter EtLsi1-1 and EtLsi6-1 and efflux Si-transporter EtLsi2-1 and EtLsi2-2 may play some roles in tef root to load Si from the rhizosphere to root cells and tissue distribution as reported for rice OsLsi1, OsLsi2, and OsLsi6, respectively (Ma et al., 2006, 2007a; Yamaji and Ma, 2011). EtLsi2-3, Etlsi2-4, Etlsi1-1, and EtLsi6-1 may distribute Si in the leaf, as reported for OsLsi2 and OsLsi6 (Yamaji et al., 2008; Ma et al., 2011). At the node, EtLsi2-1, EtLsi2-2, and EtLsi6-1 may be involved in loading Si to the spike similar to OsLsi3 and OsLis6 (Yamaji et al., 2011). EtLsi1-2 may have a role in loading Si to the inflorescence. However, the physiological function of the tef Si-transporter needs to be studied in crops or model system.

Conclusion: Silicon application improves tef grain and biomass yield by up to 100 and 45%, respectively. Both traits are economically important because tef is used as for food and forage crop. Efforts have been made to improve tef grain yield through breeding over the last five decades, yet the average yield of tef is still below 2.0 t/ha. If validated under field condition, Si is expected to double the yield potential which will have huge impact on the lives of small-scale farmers. Si application significantly increases Si concentration in the straw (1.5% of biomass), but seed Si content was extremely low (0.02%), which is not expected to affect grain quality because rice accumulates at least 10-fold more Si in the grain than tef (Ma, 2004; de Oliveira et al., 2016). However, accumulation of high levels of silica in the biomass may affect forage quality and needs to be studied. We also observed that Si decreases seed macronutrient content, which could be due to the use of Si fertilizer with Na, which might have suppressed the uptake of major minerals such as Ca, K, Mg, P, and S, or plant growth dilution. Further study is needed to screen a range of Si amendments. Because there may be a genotypic difference in Si accumulation, there is a need to screen a large panel of tef germplasm to identify high Si accumulating accession. To our knowledge, this is the first report showing the beneficial effect of Si in tef production.

## Data Availability Statement

The datasets presented in this study can be found in online repositories. The names of the repository/repositories and accession number(s) can be found in the article/Supplementary Material.

## Author Contributions

AL-O conceived the study. AL-O, BH, AS, and WG secured funding to support research activities. AL-O, BH, and AS designed the experiments. AL-O, SC, ML, and WG conducted the experiments and generated data. WG and AL-O wrote the draft. BH, AS, and ML edited the manuscript. All authors contributed to the article and approved the submitted version.

## Conflict of Interest

The authors declare that the research was conducted in the absence of any commercial or financial relationships that could be construed as a potential conflict of interest.

## Footnotes

1 http://web.expasy.org/protparam/

2 https://npsa-prabi.ibcp.fr/cgi-bin/npsa_automat.pl?page=npsa_sopma.html

3 http://www.sbg.bio.ic.ac.uk/phyre2

4 http://www.cbs.dtu.dk/services/TMHMM/

5 http://wlab.ethz.ch/protter/

6 http://meme-suite.org/tools/meme

7 bioedit.software.informer.com

## Supplementary Material

Supplementary Table 1 Sequence information of the tef Lsi proteins family identified in this study.

Supplementary Table 2 Primers of the tef putative Si transporter genes used for real-time PCR.
